# Green synthesised AuNps using *Ajuga Bracteosa* extract and AuNps-Free supernatant exhibited equivalent antibacterial and anticancerous efficacies

**DOI:** 10.1371/journal.pone.0282485

**Published:** 2023-08-07

**Authors:** Sadaf Azad Raja, Saiqa Andleeb, Aneela Javed, Sana Sabahat, Fahed Parvaiz, Hafsah Mureed, Sohaib Ahmad, Falak Naz

**Affiliations:** 1 Department of Bioscience, COMSATS University, Islamabad, Pakistan; 2 Microbial Biotechnology Laboratory, Department of Zoology, The University of Azad Jammu and Kashmir, King Abdullah Campus, Muzaffarabad, Pakistan; 3 Department of Healthcare Biotechnology, Atta-ur-Rahman School of Applied Biosciences (ASAB), National University of Sciences and Technology (NUST), Islamabad, Pakistan; Kwangwoon University, REPUBLIC OF KOREA

## Abstract

The current study is designed to synthesize gold nanoparticles using *Ajuga bracteosa* extract, which is a highly known medicinal herb found in the northern Himalayas. The synthesized gold nanoparticles were initially characterized by UV-Vis spectrophotometer, SEM, FTIR, pXRD, and, GC-MS. Antibacterial efficacy of *A*. *bracteosa* extract, AuNps, and AuNps-free supernatant activity was checked against highly pathogenic clinical isolates of *Escherichia coli*, *Staphylococcus aureus*, and *Pseudomonas aeruginosa via* agar well diffusion method, assuming that supernatant might have active compounds. The Nps-free supernatant showed the maximum antibacterial activity against *E*. *coli* (20.8±0.3 mm), *Staphylococcus aureus* (16.5±0.5), and *Pseudomonas aeruginosa* (13±0.6). While green synthesized AuNps showed effective antibacterial activity (*Escherichia coli* (16.4±0.3mm), *Staphylococcus aureus* (15.05±0.5mm), and *Pseudomonas aeruginosa* (11.07±0.6mm)) which was high compared to *A*. *bracteosa* extract. Anticancer activity was assessed by MTT assay on U87 and HEK293 cell lines. Aj-AuNps have an antigrowth effect on both the cell lines however Aj-AuNps-free supernatant which was also evaluated along with the Aj-AuNps, showed high toxicity toward HEK293 cell line compared to U87. Further, the GC-MS analysis of supernatant showed the presence of resultant toxic compounds after the reduction of gold salt, which include Trichloromethane, Propanoic acid, 2-methyl-, methyl ester, Methyl isovalerate, Pentanoic acid, 2-hydroxy-4-methyl-, Benzene-propanoic acid, and alpha-hydroxy. Based on the observation small molecular weight ligands of *Ajuga bracteosa* were analyzed in-silico for their binding efficacy towards selected membrane proteins of our target pathogens. RMSD is also calculated for the best docked protein ligand pose. The results revealed that among all listed ligands, Ergosterol and Decacetylajugrin IV have high virtuous binding affinities towards the membrane proteins of targeted pathogens. The current findings revealed that the Aj-AuNps are good antibacterial as well as anticancerous agents while the Nps-free supernatant is also exceedingly effective against resistant pathogens and cancer cell lines.

## Introduction

Metallic nanoparticles have gained enormous attraction in the field of therapeutic pharmacology. The trend of synthesizing metallic nanoparticles became more focused to keep things cost-effective and environment-friendly. To lodge these factors, green synthesis emerged as the finest technique to synthesize metallic nanoparticles, especially using medicinally known phytochemicals [1]. Phytochemicals obtained from plant extracts contain capping, reducing, and stabilizing agents that effectively synthesize metallic nanoparticles. Among metals, Gold has specific characteristics, which make it the metal of choice for most scientists. Gold nanoparticle synthesis using medicinal plant extract has been proven potential source as an antibacterial, anticancerous, antiphrastic as well as drug delivery and bioimaging therapy [2]. Green synthesis has gained attention today due to the environmentally friendly procedure and also several advanced methods of AuNp synthesis are still under the process of development and their implication in the field of medicine [3]. The chemical procedure for the synthesis of metallic nanoparticles is often toxic and might cause health issues due to the usage of reactants [4]. To avoid such incidences, green synthesis/biological synthesis has been emerged as a newer and safer technique. Among biological materials used for the synthesis of metallic nanoparticles, such as bacteria, fungus, and plant extracts, medicinal plant extracts being more effective have gained tremendous attention [5, 6]. Not only medicinal plants exhibit powerful reducing and capping abilities plus they aid an additional killing effects towards pathogens and even cancer cells [7, 8]. Medicinal plant extracts contain active compounds like phenolic acids, alkaloids, sterols, terpenoids, etc, that are very good stabilizing and reducing agents for the silver and gold nanoparticles [9, 10]. Many similar studies of synthesizing gold nanoparticles from non-medicinal plant extracts including their parts like leaf, bark, and fruit peels have also shown significant antibacterial activities [11]. Among many different reducing and capping agents, the few that are reported in multiple studies are flavonoids, phenols, and sterols [12].

Gold as a metal has many useful properties, especially its surface Plasmon characteristics based on which researchers always have been utilizing them for different therapeutic purposes, for instance, gold nanoparticles have high surface area which is very much useful for its cell permeability [13]. In addition to the high surface to volume ratio it has an excellent surface charge properties. However, green synthesized AuNps had been proved to have a less cytotoxic effect compared to citrate-stabilized AuNps [14]. Apart from antibacterial activity, AuNps have greater anticancer activity towards different cancer cells shown in in-vitro experiments, with a moderate cytotoxic effect [4]. However the AuNps are the most promising therapeutic agents in the cancer treatment.

*Ajuga bracteosa Wall Ex*. *Benth* (Neelkanthi) is a medicinal herb that is used in traditional medicine and Ayurvedic preparations. Moreover, it has been extensively used as an anti-inflammatory, analgesic, antidepressant, and anticoagulant due to the steroid components in its methanolic extracts [15]. Furthermore, *A*. *bracteosa* extracts were reported for a significant pharmacological response toward inhibiting cholinesterase enzymes in Alzheimer’s [16]. In a recent study, silver nanoparticles synthesized using plant extract of *A*. *bracteosa* have shown antimicrobial activity against bacteria like *Staphylococcus aureus*, *Salmonella typhimurium*, *Escherichia coli*, etc. [17].

The current research was designed to synthesize gold nanoparticles using extracts of aerial parts of *A*. *bracteosa* to evaluate the antibacterial and anticancerous activities of green synthesized gold nanoparticles along with the in-silico analysis of active ligands have been done.

## Materials and methods

### Extract preparation of *Ajuga bracteosa*

*A*. *bracteosa* was collected from District Muzaffarabad, Azad Jammu, and Kashmir, Pakistan. It is a medicinal plant traditionally used for several ailments, including diarrhea and dysentery [18]. Aerial parts were dried and extracts were prepared using two methods i.e. **boiling method**: Five grams of the dried crushed plant powder was dissolved in 250ml of deionized water. The resulting mixture was then boiled at 100°C, and till the volume was reduced to half. The boiled extract was filtered through grade 1 Whatman filter paper and stored at 4°C for further processing [19]. **Infusion method:** Five grams of the dried macerated plant powder were mixed with 250ml of deionized water. The mixture was kept very slow shaking at room temperature for 72 hours. The infusion prepared was then filtered through grade 1 Whatman filter paper stored at 4°C for further processing. Both extracts boiled extract (BE) and infusion extract (IE) were used to prepare AuNps [20].

### Green synthesis of gold nanoparticles

Chloroauric acid (HAuCl_4_) 1mM, was used to synthesize gold nanoparticles, preparation is as follows: 4mL of Chloroauric acid (HAuCl_4_) was added to 10mL of boiled plant extract (BE), and infusion extract (IE) separately as a standard procedure. Furthermore, the mixture was kept in a water bath at 40°C and the reaction was kept going on for the next 24 hours. Further, both of the mixtures of AuNps were centrifuged at 15000rpm for 20 minutes giving intervals of 10 minutes. The temperature was kept at 4°C. IE-AuNps were obtained in a very low amount (0.04g/14mL). Pellets of BE-AuNps (0.3g/14mL) formed at the bottom of the Eppendorf tubes were rinsed with distilled water, air-dried at room temperature (25°C), and stored at 4°C. BE-AuNps-free supernatant had a dull yellow color and was also collected and stored at 4°C. Both AuNps and AuNps-free supernatant were used for the evaluation as antibacterial and anticancer activities.

### Characterization of green synthesized AuNps

A change in the color of the mixture (Chloroauric acid solution and plant extract) was observed soon after mixing. The mixture was incubated in a water bath for 24hrs at 40°C. The color of the mixture started changing from light reddish to dark reddish brown color which indicated the synthesis of AuNps. The characterization of green synthesized AuNps was carried out using UV-visible Spectrophotometer (Shimadzu 1600). The absorbance was measured at 200 nm, 400 nm, 600 nm, and 800nm. The structural characterization of green synthesized AuNps was identified through a scanning electron microscope (SEM: Jeol JSM-6510LV). FTIR spectrometric analysis (Perkin Elmer Spectrum 100 series) was also performed for synthesized BE-AuNps only because there was no characteristic peak observed for the IE-AuNps.

### GC/MS analysis

GC-MS analysis of BE-AuNps-free supernatant was performed to identify the active compounds, using Shimadzu Qp2010T instrument, assisted with autosampler and gas-chromatography interfacing the mass-spectrometer. The conditions adjusted were as follows: helium gas (99.99%) was used as a carrier at a constant flow rate of 2mL/min with 1uL of injection volume. The capillary column used was 30m (DB-1/RTX-MS) programmed to reach a temperature of 200°C. MS parameters were adjusted to electronic ionization at 70eV. The sample analysis was determined by comparing the component’s retention times and mass weights with those of authentic samples obtained by GC-MS from the Wiley libraries and the National Institute of Standards and Technology (NIST) database.

### Antimicrobial assay of AuNps and AuNps-free Supernatant

The antibacterial efficacy of *A*. *bracteosa* extracts (Boiled extract; BE and Infusion extract; IE), green synthesized gold nanoparticles (BE-AuNps and IE-AuNps), and AuNps-free supernatant of the same (BE-AuFS and IE-AuFS) were analyzed against clinical isolates *Pseudomonas aeruginosa*, *Escherichia coli* and *Staphylococcus aureus* using agar well diffusion method [21]. The working solutions were diluted serially using 5% DMSO. The bacteria were added to a nutrient broth medium for growth and incubated for 24 h on a rotary shaker at 37°C. The incubated culture was mixed in a freshly prepared nutrient agar medium (NAM) at 45°C. The mixture was poured into sterilized Petri dishes and solidified in laminar flow at room temperature. Three wells (5 mm in diameter) in each plate were made by using a sterilized micropipette tip. In each prepared well about 10 μl of each sample were added and then placed for 24 h at 37ºC. According to Seeley et al. (2001), the growth of bacteria was determined in 24–48 h, and the diameter of the inhibition zone in mm was also measured with the help of a ruler [22, 23]. Standard antibiotics (Gentamycin, Ampicillin, and Streptomycin (10mcg concentration of each antibiotic)) were used as positive controls.

### Anticancer activity of the BE-AuNPs and BE-AuNps-free supernatant

#### Cell culture

Anticancer activity of AuNPs and supernatant was analyzed using a U87 MG cell line, derived from a human malignant glioma, and Human Embryonic Kidney 293 (HEK293), the cell line was used as a control non-cancerous cell line. The cell lines were cultured and maintained in Dulbecco modified eagle media (DMEM)–high glucose with 10% FBS (Thermo Fischer Scientific, Waltham, MA, USA) and 1% pen-strep MTT (3-[4,5-dimethylthiazol-2-yl]-2,5-diphenyltetrazolium bromide (Sigma-Aldrich, St. Louis, MO, USA). Cells were maintained and upon reaching 90 percent confluence, trypsinized and subcultured until needed for MTT assay.

#### In vitro cytotoxic assay (MTT assay)

The percentage cytotoxicity of all compounds was evaluated by dose-dependent MTT analysis. Exponentially growing cells were counted and 10,000 cells per well were plated, in triplicate, in flat-bottomed 96-well plates (Nunc, Roskilde, Denmark) for both cell lines respectively. The volume of the cells was kept at 100 μL per well. Compounds including plant extracts, AuNPs, and Supernatants were dissolved in DMEM complete media, separately, to obtain different concentrations (100/100ul, 50/150ul, and 25/175ul). Each concentration of the extract along with the original concentration was added to the 96-well plate, to obtain a final volume of ~200 μL/well. In addition, each concentration was tested in triplicate on U87 and HEK 293 cells for 72 hours. Control wells contained solvent control (without drug), standard anti-cancer drug (Doxorubicin), and blank media (without cells). Subsequently, 5 mg/mL of MTT was dissolved in 1 mL PBS. Accordingly, 15μL of prepared MTT solution was added to each well and incubated for 3 h at 37°C, so that intracellular purple formazan crystals became visible under the microscope. Following the formation of formazan crystals, all the solution from each well was removed. Then solubilizing solution, i.e., 150 μL DMSO, was added to each well. The plates were left at room temperature for a few minutes while the DMSO solution was mixed thoroughly by pipetting up and down to dissolve the formazan crystals. Finally, the absorbance of the cells was measured by a spectrophotometer at 550 nm.

The influence of the formulations on the cell viability was determined using the following formula:

$$\%\;viability=\left(A570\;of\;treated\;cells-A570\;of\;blank\;cells\right)/\left(A570\;of\;controlled\;cells-A570\;of\;blank\;cells\right)\times 100.$$

#### Molecular docking of active Ligands with vital membrane proteins of selected pathogens

The binding of a ligand to its respective target protein causes alteration in the shape and/or activity of the ligand [24]. The 2D structures of ligands were downloaded from PubChem in SDF format to get multiple conformational structures in a single file for getting the best-docked ligand conformation. Docking is the most advanced computational method in structure-based drug designing to obtain precise conformation of ligand-receptor interaction and to analyze their relative orientation [25]. To find out the potential antibacterial activity, docking of *A*. *bracteosa* active ligands with Penicillin Binding Protein 2a –PBP2a (PDB ID: 5M18) and Capsular Polysaccharide Biosynthesis Protein A—CapA (PDB ID: 4JMP) of *S*. *Aureus*, Lipopolysaccharide Transfer Protein D—LptD (PDB ID: 2M7I) and β-Barrel Assembly Machinery B- BamB (PDB ID: 4HDJ) of *P*. *aeruginosa*, and, Penicillin Binding Protein 1B –PBP1b (PDB Id: 3FWL) and β-Barrel Assembly Machinery A- BamA (PDB ID: 4M75) of *E*.*coli* was done. For this purpose, AutoDock Vina was used which is a molecular modeling simulation software and is especially effective for protein-ligand docking. For the final PDB preparation of the target proteins the structures were purified. Water residues and unwanted ligands from the structures of proteins were removed, and polar hydrogen atoms and Kollman charges were also added to the protein structures. Active/target sites of proteins were unknown, for that reason whole protein was selected in the grid dimension file [26]. The configuration files of the target proteins generated the ten best conformations for each ligand. The best conformation of each ligand with selected protein was analyzed using Biovia Discovery Studio.

### Finding out binding affinities

Ligand-receptor interactions indicate the occurrence of physiological processes and the strength of these interactions is identified by binding affinity. Binding affinity is the basic output generated after docking [27]. Binding affinities were calculated for each selected pathogen protein and *A*. *bracteosa* ligands. The protein-receptor complexes having considerable binding affinities were further analyzed. Binding pockets and interacting residues of the protein-ligand complex were identified using Biovia Discovery Studio. The type of bonds formed between them was also identified.

### Statistical analysis

Statistical analyses were performed using GraphPad Prism 6 (GraphPad Software, Inc.). Frequency distribution test was performed and overlaid by the Gaussian test for the standard curve to obtain the mean particle size. The mean and or average inhibitory zone produced by targeting agents is calculated via a simple statistical analysis of the experiment run in triplicates. Wilcoxon test was performed for calculating variance in the anticancer activity of the targeting agents.

## Results and discussion

### Initial characterization *A*. *bracteosa* extract and green synthesized AuNps

**Fig 1:**
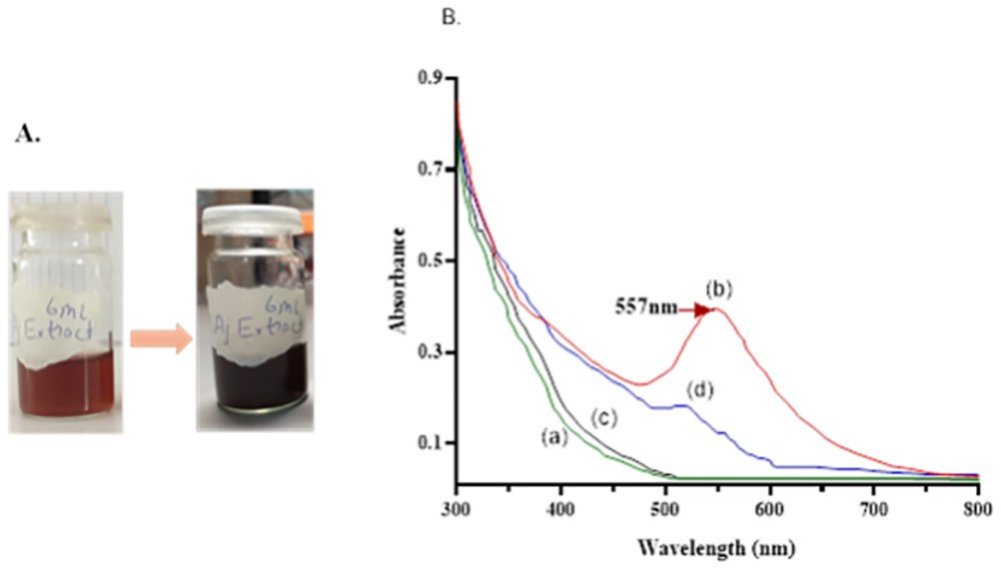
Colour change during green synthesis of AuNps synthesized from boiling extract. Absorption spectra of BE-AuNps and IE-AuNps are shown as (b & d). The boiling extract and infusion extract absorption spectrum is shown as (a & c). The sharp peak was observed at 557nm indicating gold nanoparticle synthesis (b).

**Fig 2:**
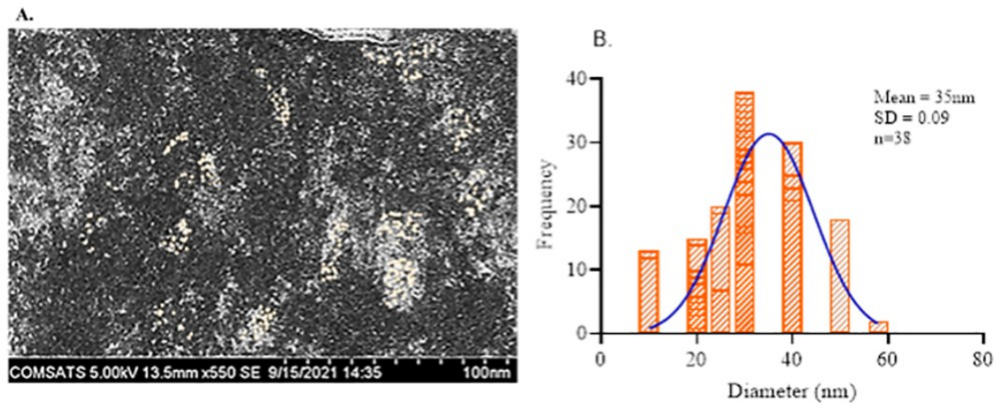
A. SEM image of the green synthesized Aj-AuNps at the resolution of 100nm. (B) The particle size was determined in the histogram overlaid by the Gaussian curve showing an average diameter of 35nm.

The A. *bracteosa* extract has already been characterized through Thin Layer Chromatography for phytochemical screening and reported in our recent work [17]. Previous studies revealed the presence of saponins, tannins, alkaloids, free amino acids, quinones, phenols, steroids, glycosides, terpenoids, and flavonoids in an aqueous extract of *A*. *bracteosa* [17, 20]. Initial characterization using UV-Vis Spectrophotometer was done for plant extracts prepared through boiling BE and infusion IE and the respective AuNps. Results revealed that the color of the mixtures started changing from light reddish to dark reddish-brown color in the case of BE-made AuNps (Fig 1A). The nanoparticle syntheses were seen very low in IE-made AuNps, indicated by no obvious change in color. Absorption spectra of boiling plant extract and infusion plant extract are shown in Fig 1B (1a & 1c) respectively. Synthesis of AuNPs was confirmed *via* UV-Vis spectrophotometry. The absorption peak was observed at 557nm in BE-made AuNps (Fig 1B (b)). There was no apparent absorption peak observed in the mixture of infusion extract and gold salt solution (Fig 1B (d)). However, a minor shift of absorbance was detected in IE-made AuNps at 530nm. Infusion plant extract might lack appropriate capping and reducing agents needed to synthesize gold nanoparticles. The minor shift of absorbance might indicate aggregates of nanoparticles might be present in IE-AuNps. Whereas the boiling extract contained the important required reducing capping and stabilizing phytochemicals henceforward significantly made the gold nanoparticles (Aj-AuNps). Based on the initial characterization of the mixtures through Uv-Vis Spectrophotometry the Scanning Electron Microscopy of the only green synthesized Aj-AuNps was carried out at 100nm. The average particle size synthesized was calculated as equal to 35nm±0.09nm using ImageJ software (Fig 2a). The size determined corresponds to many different studies in which plant extracts and dried parts have been used for the synthesis of gold nanoparticles. The size range of green synthesized gold nanoparticles is between 5nm to around 70nm [13, 26–30]. The size range and frequency distribution are given in the graph (Fig 2b). the average size of the particle was comparatively larger than indicating the stabilizing capacity of the capping agents of the boiling extract.

### Fourier transform infrared spectroscopy

**Fig 3:**
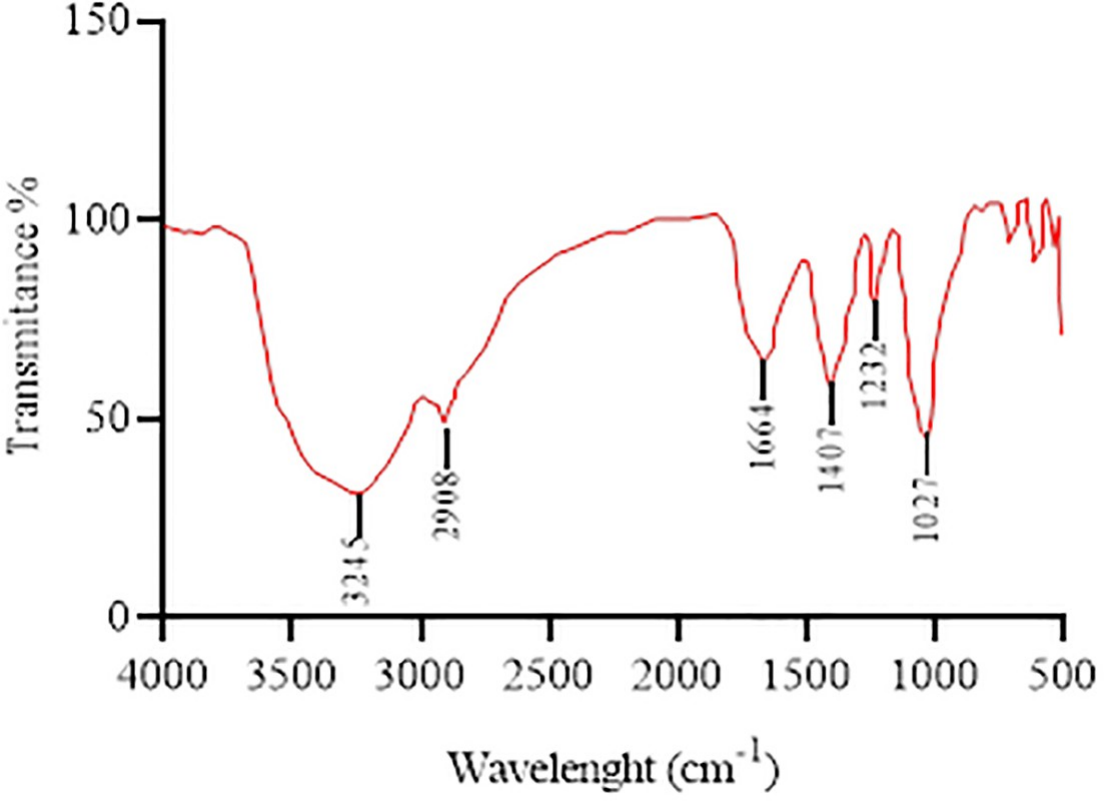
FTIR spectrum of the green synthesized Aj-AuNps.

Fourier Transform Infrared Spectroscopy (FTIR) analysis was carried out for the green synthesized AuNps from boiling plant extract only to determine the possible functional groups responsible for capping, stabilizing, and reduction of gold nanoparticles. There were no gold nanoparticles synthesized from the infusion extract (Fig 1B (b)). The IR spectrum of synthesized Aj-AuNps showed the major stretching between 3000 to 3500 cm^-1^ which corresponds to the O-H stretch and hence indicates the presence of alcohols, phenols, and flavonoids. Another minor stretch that appeared at 2908 cm^-1^ signifies the presence of a C-H bond that indicate the involvement of alkanes. Further down the C = O stretching was observed at 1664 cm^-1^, showing carbonyls in general. Transmittance between the region 1500–1400 cm^-1^ indicated aromatic C-C stretch which corresponds to the aromatic compound phenol like Ajuganane, a newly discovered phytochemical found in *A*. *Bracteosa* extracts [31]. In last the C-O-C bond stretch was observed within the range of 1400–1000 cm^-1^ (Fig 3). The IR results exposed the active compounds involved in the reduction, capping, and stabilizing of gold nanoparticles and they might be flavonoids, phenols, and proteins contained in the extract are usually responsible for capping and other phytocompounds like terpenes could be responsible for their stability. These compounds in various plant extracts have already been reported as effective gold nanoparticle synthesizing agents [29].

### X-ray diffraction analysis of green synthesized AuNps

**Fig 4:**
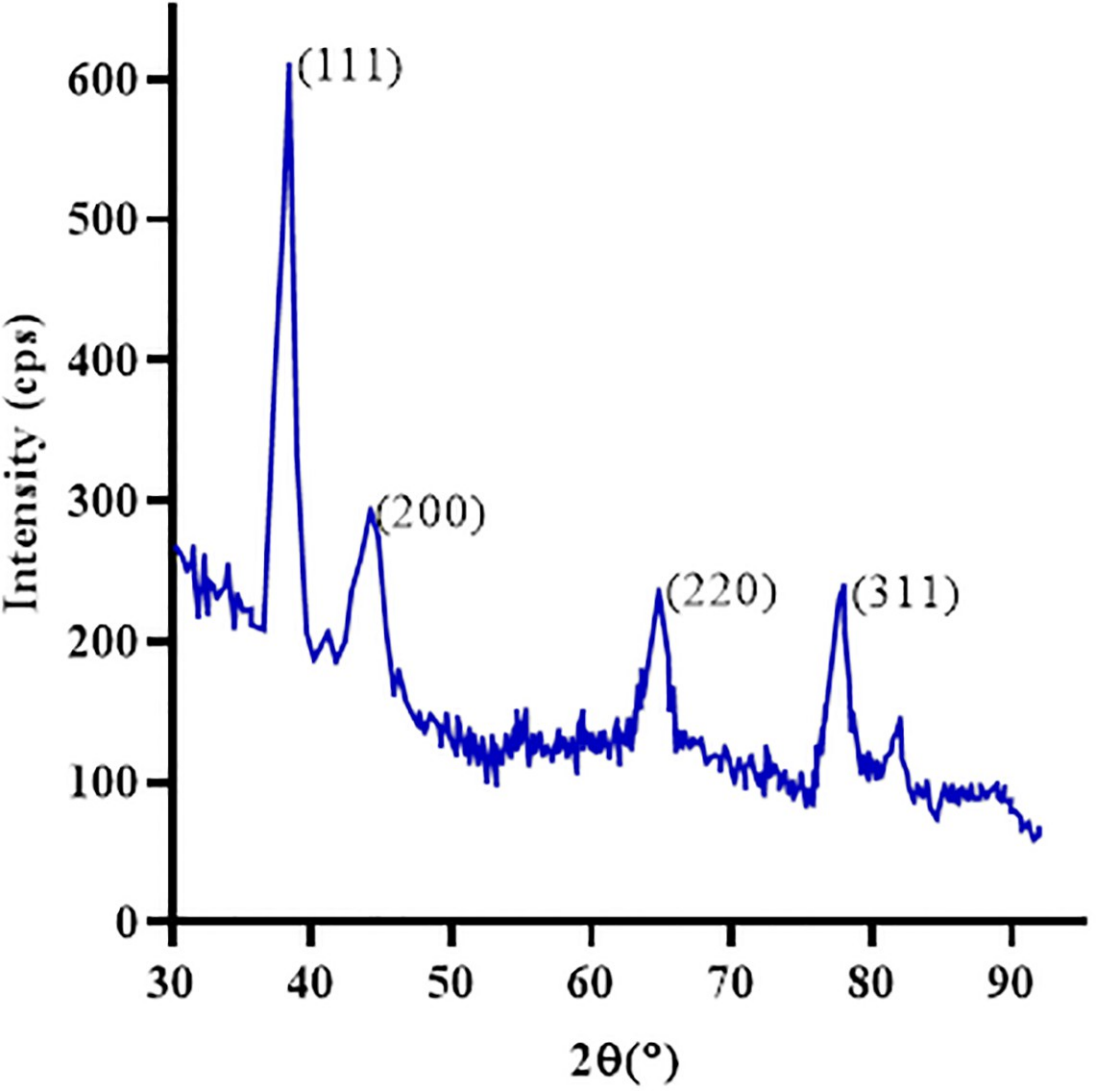
The pXRD spectra of Au-Nps synthesized from Ajuga *bracteosa* plant extracts.

The green synthesized AuNps were analyzed for their crystalline structure through a Power X-ray diffraction pattern (Fig 4). The peak pattern shows the synthesized crystalline structure of AuNps. The spectrum gives an intense peak at 2*θ* = 38.38°, 44.19°, 64.75°, and 78.02° which corresponds to the (111), (200), (220), and (311) Bragg’s reflections of face centre cubic (fcc) plane of AuNps crystalline structure according to JCPDS database (JCPDS file number 00-004-0784) [11]. The particle size of Au-NPs was calculated using the Debye-Scherrer equation (D = Kλ/β.cosθ) which was around 16nm.

### GC-MS analysis of BPE-Aj-AuNps free supernatant

**Fig 5:**
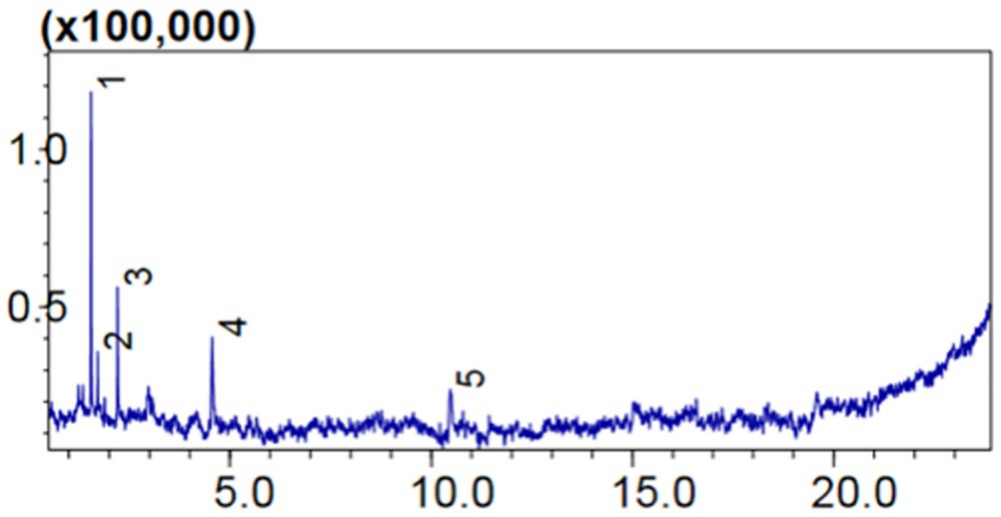
GC-MS chromatogram of Aj-AuNps-free supernatant.

The qualitative phytochemical analysis through GC-MS of the selected medicinal plant, *A*. *bracteosa* has already been reported. The phytochemicals found were alkaloids, phenolic, tannins, cardiac glycosides, terpenes, flavonoids, saponins, steroids, carbohydrates, proteins, polysterols, and amino acids [32]. GC-MS analysis was also reported for the presence of bioactive compounds. More than fifty bioactive compounds have been identified through GC-MS analysis of ethyl acetate fraction of *A*. *bracteosa* ranging from the molecular weight of 92 to 278 [11, 32]. In our study, the antibacterial activity was found different. The bacterial growth inhibition was seen maximum in those experiments where the selected pathogens were treated with Aj-AuNps free supernatant. The BE-AuNps-free solution was thought to be evaluated as if certain toxic compounds were present in the supernatant solution after pelleting of AuNps. The results stood positive via antibacterial efficacy analysis. Therefore, the Aj-AuNps-free solution was subjected to its GC-MS to identify the phytochemical components present which is responsible for antimicrobial activity. The chromatogram showed five major peaks (Fig 5). The aspects of analysis with the corresponding Phyto component are shown in Table 1. The supplementary file is also given (S1 File).

**Table 1:** Phyto-components identified in the Aj-AuNps-free supernatant by GC-MS.

Serial #	Name of the compound	Retention Time	Area %	Molecular formula	Molecular weight
1	Trichloromethane	1.550	38.63	CHCl_3_	118
2	Propanoic acid, 2-methyl-, methyl ester	1.716	6.52	C_5_H_10_O_2_	102
3	Methyl isovalerate	2.211	16.97	C_6_H_12_O_2_	116
4	Pentanoic acid, 2-hydroxy-4-methyl-, met	4.556	22.67	C_7_H_14_O_3_	146
5	Benzene-propanoic acid, alpha-hydroxy	10.469	15.22	C_10_H_12_O_3_	180

The first observable peak indicated Trichloromethane which is a known toxic compound used as an anesthetic drug (PubChem Compound Summary for CID 6212). Its presence in the BE-AuNps has strongly affected microbial growth. Similarly, other compounds identified as per the peak shown in Table 1 are propanoic acid, the second peak is a strong antioxidant and immunosuppressant. Might be it has no specific impact on microbial growth but could be directly responsible for the growth inhibition of human cancer cells. Propanoic acid has many beneficial effects on human metabolism and it is a good anti-inflammatory compound [33, 34]. Likewise, Pentanoic acid, the 3^rd^ peak, is an HDAC8 (Histone deacetylase 8) inhibitor [35]. The fourth compound identified was methyl Isovalerate which is a known supplemental for the small intestine development in growing calves [36]. Overall, the efficacy of BE-AuNps-free supernatant was found strong and the presence of the above-mentioned chemicals is the bases of its activity against bacterial pathogens.

#### Bioinformatics’ analysis of active ligands

The activity of AuNps-free supernatant was further excavated through *in-silico* analysis. There are almost 73 active ligands identified to date in *A*. *bracteosa* extract, which were listed from text mining of different research papers (Table 2). The structure of these ligands was downloaded from PubChem in PDF format. For the ligands having unknown structures, the structures were drawn on the Reactor tool and downloaded in PDF format. These files were converted into PDB format using Bio via Discovery Studio. To identify small ligands, the molecular weights of these ligands were determined with the help of the Reactor tool. *A*. *bracteosa* ligands’ molecular weight ranged from 136g/mol to 934g/mol, from which the ligands having a molecular weight up to 396g/mol were shortlisted as we needed the most likely ligands in the AuNps-free supernatant. We performed the in-silico analysis of the effectiveness of these shortlisted ligands against the important bacterial membrane proteins.

**Table 2:** Active ligands of *A*. *bracteosa* identified to date.

Active ligands of *A*. *bracteosa*	
1. Limonene	2. β-Myrcene
3. Camphene	4. α-phellendrene
5. Perilla alcohol	6. linalyl acetate
7. α-humulene	8. βCaryophellene
9. Elemol	10. Palmitic acid
11. Linoleic acid	12. Oleic acid
13. Ajuganane	14. deacetylAjugarin IV
15. 3,4′-Dihydroxy-3,6,7trimethoxyflavone	16. lignoceric acid
17. 7-Hydroxy-3,6,3′,4′tetramethoxyflavone	18. Ceryl alcohol
19. Ajugarin V	20. Ajugarin II
21. Reptoside	22. Cerotic acid
23. Heptacos-3-en-25-one	24. Bis(2S-methylheptyl) phthalate
25. Ergosterol	26. Ajugarin IV
27. 8-o-acetylharpagide	28. Bracteonin A
29. Stigmasterol	30. Ergosterol 5,8- endoperoxide
31. β- Sitosterol	32. Amphotericin B
33. Ajugarin I	34. Clerodin
35. dihydroclerodin-1	36. Dihydroclerodin
37. Ajubractin E	38. Ajugarin III
39. Ursolic Acid	40. 14-hydro -15- hydroxyclerodin
41. Clerodinin A	42. Ajugin
43. Withaferin-A	44. Ajugasterone C
45. 20-hydroxyecdysone	46. 24, 25-epoxywithanolide D
47. 3-epi-Caryoptin	48. 3-epi-14,15-Dihydrocaryoptin
49. Ajugasterone A	50. 12-Bromo-Ajugarin I
51. Bracteosin c	52. Bracteosin A
53. Ajugasterone B	54. Ajugalactone
55. Ajubractin B	56. Cyasterone
57. 3-Epicyasterone	58. Ivan II
59. Physangulide	60. Ajubractin A
61. Bracteosin B	62. Ajubractin D
63. Ajubractin C	64. Ajugapitin
65. 14-Hydro-15-hydroxyajugachin A	66. 14, 15-dihydroajugapitin
67. 15-Hydroxyajubractin C	68. Sitoglucoside
69. 22-Acetylcyasterone	70. 15-epi-Lupulin B
71. Bractin A	72. Bractic acid
73. Bractin B	

#### Selection of target proteins

**Fig 6:**
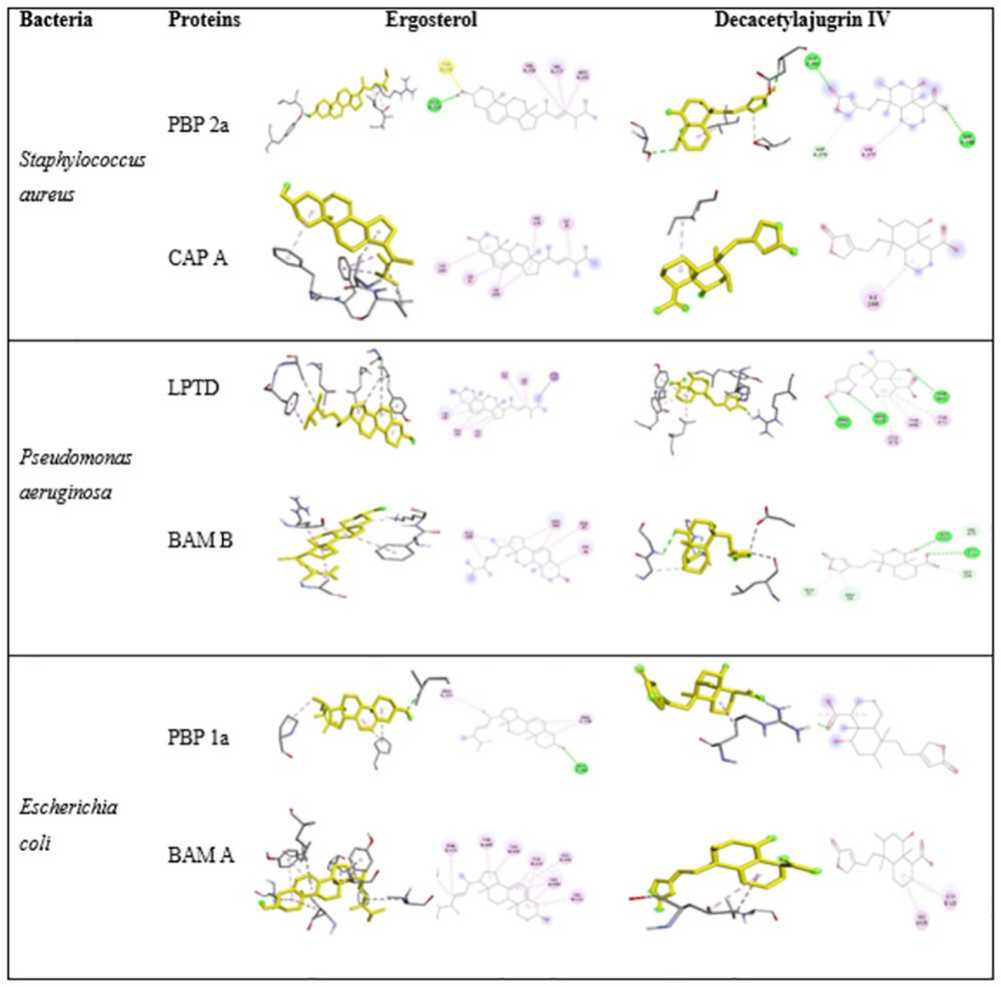
Ligand binding pockets are shown interacting with the selected target proteins of the respective bacteria. Ligands are shown in yellow color. Conventional hydrogen bond is shown in green color, carbon-hydrogen bond is shown in sky blue, Pi-Anion in orange and Alkyl bonding is shown in pink color.

The membrane proteins of bacterial pathogens have been known to play important role in their survival, protection, growth, and resistance. A few membrane proteins of *Escherichia coli*, *Staphylococcus aureus*, and *Pseudomonas aeruginosa* have been selected as target proteins, their important function is mentioned against each selected proteins in Table 3. The binding affinities of the small ligands with bacterial target proteins are shown in Table 4. Based on the highest ligand affinity two ligands were further selected, that are Ergosterol and Decacetylajugrin IV to identify their bonding strength (Fig 6) and the RMSD was also calculated using Autodock Vina [37] (Table 5). The binding pockets revealed the strong binding of ligands to their targeted proteins through conventional hydrogen bonds which are the strongest of hydrogen bonds [38].

**Table 3:** Selected membrane proteins of the target bacterial isolates and the relevant information. The data is collected from Uniport.

Organism	Protein	Function	Length a.acids	PDB ID	Uniprot ID
*Staphylococcus aureus*	PBP2a	The final step of bacterial cell wall synthesis	668	5M18	Q7DHH4
	CapA	Biosynthesis of type 1 capsular polysaccharide	221	4JMP	P39850
*Pseudomonas aeruginosa*	LptD	Assembly of lipopolysaccharide in the outer cell membrane	927	2M7I	A0A485FR X1
	BamB	Assembly and insertion of beta-barrel proteins into the outer membrane.	380	4HDJ	A0A072Z QH3
*Escherichia coli*	PBP1b	Cell wall biogenesis	804	3FWL	P02919
	BamA	Assembly and insertion of beta barrel proteins in the outer membrane	810	4M75	P0A940

**Table 4:** Binding affinities of ligands and bacterial proteins.

Organisms	*Staphylococcus aureus*		*Pseudomonas aeruginosa*		*Escherichia coli*	
Ligands	PBP 2A	Cap A	LPTD	Bam B	PBP 1B	Bam A
1. Limonene	-6.0	-5.2	-6.0	-4.7	-6.1	-6.8
2. β-Myrcene	-5.2	-5.0	-5.5	-4.6	-4.9	-5.8
3. Camphene	-5.9	-5.7	-5.8	-5.5	-7.0	-6.6
4. Alpha Phellandrene	-6.1	-5.1	-6.2	-5.4	-6.1	-6.8
5. Perilla Alcohol	-5.6	-4.5	-5.9	-5.2	-5.9	-6.1
6. Alpha Humulene	-7.3	-6.7	-7.6	-6.3	-6.7	-8.6
7. Beta caryophyllene	-7.1	-5.7	-7.1	-5.5	-6.0	-8.4
8. Elemol	-6.6	-5.5	-6.1	-4.7	-5.6	-7.0
9. Palmitic Acid	-4.5	-4.2	-4.1	-2.8	-3.1	-5.5
10. Linoleic Acid	-4.2	-4.1	-5.1	-3.8	-3.7	-5.3
11. Oliec Acid	-4.4	-3.5	-4.4	-3.9	-3.8	-5.2
12. Ajuganane]	-5.7	-5.1	-6.4	-4.2	-4.7	-7.1
13. Deacetylajugarin IV	-7.7	-5.8	-7.9	-8.0	-6.6	-9.0
14. 3,4′-Dihydroxy-3,6,7trimethoxyflavone	-7.7	-5.9	-7.0	-7.0	-7.9	-8.1
15. Lignoceric acid	-4.0	-3.0	-4.1	-3.8	-4.8	-4.3
16. 7-Hydroxy-3,6,3′,4′tetramethoxyflavone	-7.4	-5.7	-7.4	-6.4	-7.5	-8.3
17. Ceryl Alcohol	-4.4	-3.1	-3.7	-2.7	-2.9	-5.9
18. Heptacos-3-en-25one	-4.2	-2.9	-4.1	-3.3	-3.7	-5.7
19. Cerotic acid	-3.8	-3.8	-4.3	-3.3	-4.1	-4.1
20. Ergosterol	-8.8	-7.2	-7.6	-6.3	-9.1	-11.8

**Table 5:** RMSD values with energy calculations.

Target Protein	Deacetylajugarin IV				Ergosterol			
	Mode | affinity | dist. from best mode				Mode | affinity | dist. from best mode			
	| (kcal/mol) | RMSD l.b. | RMSD u.b.				| (kcal/mol) | RMSD l.b. | RMSD u.b.			
	-----+------------+---------- +----------				-----+------------+---------- +----------			
BamA	1	-7.4	0.000	0.000	1	-10.6	0.000	0.000
	2	-7.3	20.894	22.182	2	-10.2	2.622	4.348
	3	-7.3	19.547	21.984	3	-9.9	3.425	10.613
	4	-7.1	17.532	19.384	4	-9.9	3.719	7.339
	5	-7.0	18.026	19.615	5	-9.8	3.476	9.419
	6	-7.0	17.826	20.338	6	-9.8	1.830	3.618
	7	-6.9	18.963	20.137	7	-9.2	5.690	9.780
	8	-6.8	6.229	11.540	8	-8.4	20.478	22.874
	9	-6.6	18.179	19.810	9	-8.4	20.107	23.959
BamB	1	-7.0	0.000	0.000	1	-8.1	0.000	0.000
	2	-7.0	2.960	6.460	2	-7.9	3.761	9.072
	3	-6.9	2.824	5.614	3	-7.8	4.607	7.752
	4	-6.8	16.046	18.757	4	-7.6	1.323	3.009
	5	-6.8	11.284	16.991	5	-7.5	5.724	9.129
	6	-6.5	14.587	18.953	6	-7.2	3.315	8.438
	7	-6.4	3.670	5.362	7	-7.1	16.790	18.670
	8	-6.4	3.637	5.761	8	-7.0	17.322	20.103
	9	-6.3	15.662	19.850	9	-6.8	4.527	9.593
CapA	1	-5.8	0.000	0.000	1	-6.3	0.000	0.000
	2	-4.8	2.089	6.035	2	-5.5	16.986	19.301
	3	-4.5	2.956	5.419	3	-5.5	26.023	28.825
	4	-4.4	1.859	2.743	4	-5.3	9.283	10.769
	5	-4.4	29.109	31.618	5	-5.3	17.236	19.873
	6	-4.4	59.980	63.001	6	-5.2	23.314	27.822
	7	-4.3	27.829	29.741	7	-5.1	45.670	50.174
	8	-4.3	14.598	17.652	8	-5.1	14.197	17.100
	9	-4.2	36.158	38.530	9	-4.8	34.408	37.133
LptD	1	-7.6	0.000	0.000	1	-10.5	0.000	0.000
	2	-7.5	3.283	5.562	2	-9.5	1.733	2.304
	3	-7.5	2.526	6.758	3	-8.2	2.809	3.889
	4	-7.4	2.548	4.733	4	-8.0	35.014	37.029
	5	-7.2	2.219	5.961	5	-7.7	26.760	29.521
	6	-7.2	2.024	2.967	6	-7.5	35.163	36.296
	7	-7.0	44.941	48.459	7	-7.5	20.321	24.968
	8	-6.9	1.661	2.590	8	-7.4	34.659	37.430
	9	-6.5	2.686	4.108	9	-7.4	4.186	7.906
PBP1A	1	-7.1	0.000	0.000	1	-6.9	0.000	0.000
	2	-6.5	2.170	6.707	2	-6.5	41.922	47.637
	3	-6.4	2.277	2.872	3	-6.4	57.658	63.595
	4	-6.1	30.316	33.430	4	-6.3	33.430	38.770
	5	-6.1	53.719	57.069	5	-6.1	25.261	27.476
	6	-6.0	18.713	21.000	6	-6.1	53.288	59.817
	7	-6.0	39.319	41.989	7	-6.0	33.921	37.822
	8	-6.0	31.093	33.115	8	-6.0	31.136	36.589
	9	-5.9	20.707	23.098	9	-5.8	33.229	37.112
PBP2A	1	-7.4	0.000	0.000	1	-8.7	0.000	0.000
	2	-7.1	50.239	52.133	2	-8.1	1.132	2.591
	3	-6.8	52.095	53.801	3	-7.7	1.865	3.738
	4	-6.5	51.883	53.614	4	-7.6	34.102	36.051
	5	-6.5	25.595	29.576	5	-7.4	64.389	70.157
	6	-6.4	29.255	33.446	6	-7.2	61.947	67.905
	7	-6.4	39.426	42.788	7	-7.1	43.741	51.143
	8	-6.3	2.035	4.302	8	-7.1	12.808	14.719
	9	-6.3	31.686	34.086	9	-7.0	36.030	43.979

RMSD = Root Mean Square Deviation; l.b = Lower bound; u.b = Upper bound

### Antibacterial efficacy

Results revealed that green synthesized gold nanoparticles i.e. IE-AuNps and BE-AuNps showed the maximum inhibition of all tested bacterial pathogens at 0.04 mg/ml concentration (Table 6). The zone of inhibition was recorded as *Pseudomonas aeruginosa* (9.05±0.1 mm and 11.0±0.6 mm), *Escherichia coli* (11.0±0.5 mm and 16.4±0.3 mm), and *Staphylococcus aureus* (10.0±0.3 mm and 15.5±0.5 mm). Similarly, the boiled extract (BE) indicated the maximum inhibition of all bacterial pathogens with 11.0±0.3 mm, 11.2±0.3 mm, and 10.06±0.5 mm zone of inhibition while infusion extract (IE) had no antibacterial effect at 0.1 mg/ml concentration. Interesting results were recorded when AuNps-free supernatant (BE-AuFS and IE-AuFS) were applied, and it was observed that BE-AuFS represented the maximum inhibition of bacteria with 20.8±0.3 mm, 16.5±0.5 mm, 13.0±0.6 mm zone of inhibition against *Escherichia coli*, *Staphylococcus aureus* and *Pseudomonas aeruginosa* respectively, compared to IE-Au-soln. which did not affect the growth of *Escherichia coli* and *Staphylococcus aureus*. Overall, the findings revealed that Aj-AuFS has given the most efficient bactericidal effect. DMSO was used as a negative control and has shown no effect on the growth of pathogens (Table 6). On the other hand, the antibacterial efficacy of used antibiotics was recorded as 12.02±0.3 (Gentamicin), 13.04±0.5 (Ampicillin), and 11.01±0.5 (Streptomycin).

**Table 6:** Zone of inhibition in mm of plant extract (BPE and PEI), green synthesized gold nanoparticles, AuNps, and AuNps-free supernatant.

Working	5% DMSO	IE 0.1mg/ml	IE-Au-soln. 0.04mg/ml	Aj-Au-FS	BE 0.1mg/ml	Aj-AuNps 0.04mg/ml
soln. Strains						
*Escherichia coli*	-	-	11.0±0.5	20.8±0.3	11±0.3	16.4±0.3
*Staphylococcus aureus*	-	-	10.0±0.3	16.5±0.5	11.2±0.3	15.05±0.5
	-	-				-
*Pseudomonas aeruginosa*	-	-	9.05±0.1	13±0.6	10.06±0.5	11.07±0.6

Growth inhibition was recorded as 0 for resistant; >1–5 for low sensitivity; >5–10 for moderate sensitivity; >10 to 30 for high sensitivity

Previous studies reported the several biomedical applications of gold nanoparticles as used in disease diagnostics and treatments, drug deliveries, sensors development, and imaging of tumor cells, etc [39–42]. Our findings showed that green synthesized gold nanoparticles (Aj-AuNps) and AuNps-free supernatant (Aj-AuFS) had an effective antibacterial activity against both gram-positive and gram-negative bacteria. The ligands such as Ergosterol and Deacetylajugarin IV found in the *A*. *bracteosa* showed the maximum binding affinities with the plasma membrane and cell wall-associated proteins of the targeted bacterial pathogens i.e., CapA, LptD, BamB, BamA, PBP1b, and PBP2a, respectively and blocked their all activities such as the synthesis of peptidoglycan during cell proliferation, capsule synthesis, lipopolysaccharides, and beta-barrel proteins assemblage in the cell membrane, and cell elongation, leading to cell death [43–45].

Ergosterol gave the highest binding affinity with the membrane proteins of *S*. *aureus* and *E*. *coli* and consequently block their activity in the cell, decreasing the growth and proliferation rate and eventually leading to the death of the cell. Moreover, if CapA is blocked by nanoparticle/ drug, *S*. *aureus* would be unable to synthesize capsular polysaccharides and consequently will fail to evade human immune defense. Binding energy with PBP2A is -8.8 KJ/mol, Cap A is -7.2 KJ/mol, with BamA is -9.1 KJ/mol, and PBP1B is -11.8 KJ/mol. As the cell wall is important for survival and resistance under osmotic stress conditions that bacteria often come across in natural environments, including soil and the intestinal tract. Hence, the loss of function of PBP1b will cause the bacteria to become more sensitive to a hypertonic culture medium [46]. Similarly, outer membrane protein synthesis and transport are essential to maintain the functionality of the bacterial outer membrane. Outer membranes act as a defensive physical barrier and at the same time enable the transmembrane trafficking of nutrients and signaling molecules. Therefore, the proper targeting, folding, and insertion of a variety of outer membrane proteins are essential for the viability of bacteria [47]. If Bam A is blocked, the cell will lose its viability.

On the other hand, Deacetylajugarin IV showed the best docking and binding affinity with both LptD (-7.9 KJ/mol) and Bam B (-8.0 KJ/mol). When these proteins are blocked, the lipopolysaccharides to the outer membrane will not be transported. Consequently, the bacteria will lose its membrane integrity and will be open to external chemical attacks. In addition, if Bam B gets blocked by the ligand, the biogenesis of outer membrane proteins will not undergo completion. As a result, the bacteria will not be able to transport nutrients from the outside to the inside of the cell, also the signal transduction will be terminated.

The current findings agreed with the outcomes of previous studies that nanoparticles were lethal to bacterial cells [48]. It was also observed that the bactericidal effect of green synthesized gold nanoparticles is dependent on the dose, the particle size, and surface area: volume ratio, and preparation method, and our results agreed with the findings of [17]. According to [49, 50] nanoparticles are relatively small in size compared to bacterial cells and permit them to enter the cell easily. The findings revealed that the BE-AuNps or BE-AuFS exhibit potent bactericidal properties.

### Anti-cancer activity

**Fig 7:**
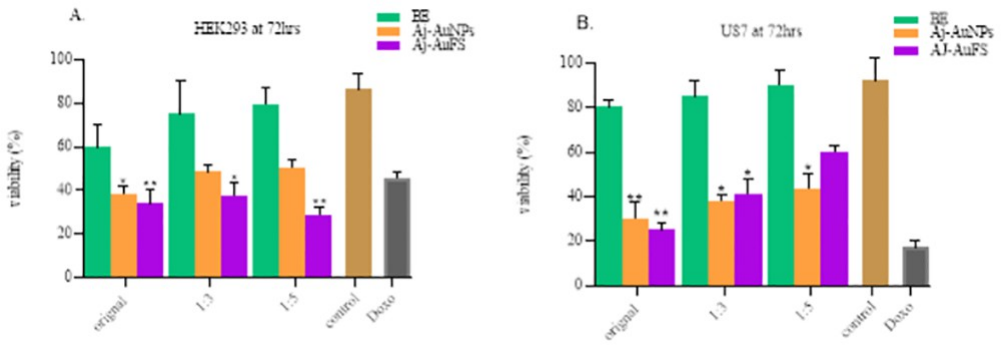
Cell viability was determined through MTT assay on human embryonic kidney HEK293 (control normal cells) and human glioblastoma cells U87. Original extract, 1:3 dilution, 1:5 dilution, control (cell media only), Doxo is (doxorubicin). (A) HEK293 cells were treated with boiled plant extract, BE-AuNps, and BE-AuNps-free supernatant at different concentrations the growth inhibition was seen significantly decreased in supernatant treatment at 1:5 concentration only (P* = 0.*01*). (B) U87 cells were also treated with the same solutions as HEK293, and a significant decrease in cell viability was observed in BE-AuNps (P** = 0.*003*) and supernatant treatment (P* = 0.*02*) at 1:5 dilution.

It was ruled out that the inhibitory growth effect of infusion extract, IE-Au-solutio, and their supernatant was not observable, while of the boiled extract, BE-AuNps and their supernatant were high, henceforth, the cell growth inhibition and or lethality/cytotoxicity is analyzed in U-87 MG cell line, derived from a human malignant glioma and Human Embryonic Kidney 293 (HEK293), the cell line was used as control non-cancerous cell line. The results were striking. The boiled plant extract (BE) showed no significant decrease in viability of U87 and control normal cells (HEK293) compared to cells treated with Aj-AuNps and Aj-AuNps free supernatant. In U87 cells the viability has significantly decreased (*P = 0*.*0034*) in cells treated with Aj-AuNps whereas, a moderate decrease in viability was found in cells treated with Aj-AuNps free supernatant (*P = 0*.*02*) (Fig 7A & 7B).

These differential results showed that the pure plant extract does not affect significant growth inhibition in both cell lines. Whereas the Aj-AuNps and Aj-AuFS showed a dose-dependent cytotoxicity activity in both HEK and U87 cell lines. The effect of both formulations on cytotoxicity of the U87 cancer cell line was found to be more pronounced (Aj-AuNps all dilutions; 38.8%, 40.6%, 50.3% Vs 30.2%, 38.01%, 43.1%, BE- AuFS; 34.1%, 37.1%, 28.6% Vs 25.2%, 41.3%, 60.2%) as compared to HEK cells. The trends are similar to the standard anticancer drug, doxorubicin indicating that the formulations are differentially cytotoxic towards the cancer cells as compared to normal cells. Overall, the effect of Aj-AuNps was observed non-significantly different from the doxorubicin a standard anticancer drug. This shows that the Aj-AuNps can be an efficient anticancer nanoparticle and are environmentally friendly and less toxic to normal cells. Phytochemicals present in the BE-AuFS had a very contrasting result that is might be due to the protein expressional and plasma membrane properties change in the U87 cells.

## Conclusion

The green synthesized gold nanoparticles and their AuNps-free supernatants have shown significantly good growth inhibition of selected bacterial pathogens as well as cancer cells in the in-vitro analysis compared to the crude aqueous extract of *A*. *bracteosa*. The Aj-AuNps-free supernatant being very toxic to bacteria and normal human cells inhibits moderately the growth of cancer cells (U87 MG) which might be due to the differential protein expression of cancer cells making them resistant. The synthesized nanoparticles and their free supernatant needed to be further evaluated for their activity. In conclusion, Aj-AuNps can be obtainable as a good antimicrobial and or anticancer agent along with the byproducts in the supernatant that could serve as good candidates for antimicrobial compounds.

## Supporting information

S1 File (XPS)
