# Supplementary figures and images for: Green synthesised AuNps using Ajuga Bracteosa extract and AuNps-Free supernatant exhibited equivalent antibacterial and anticancerous efficacies
Source: PLoS One. 2023 Aug 7;18(8):e0282485. doi: 10.1371/journal.pone.0282485 (PMC10406282; doi:10.1371/journal.pone.0282485)

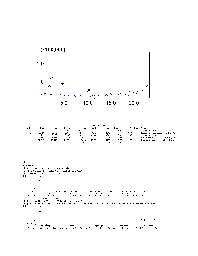

Supplement: S1 File — (XPS) [file pone.0282485.s001.xps › Documents/1/Metadata/Page1_Thumbnail.JPG]
